# New products

**DOI:** 10.1155/S1463924697000138

**Published:** 1997


Journal of Automatic Chemistry, Vol. 19, No. 4 (July–August 1997) pp. 141–144

### New products

### Fast data acquisition from your PC

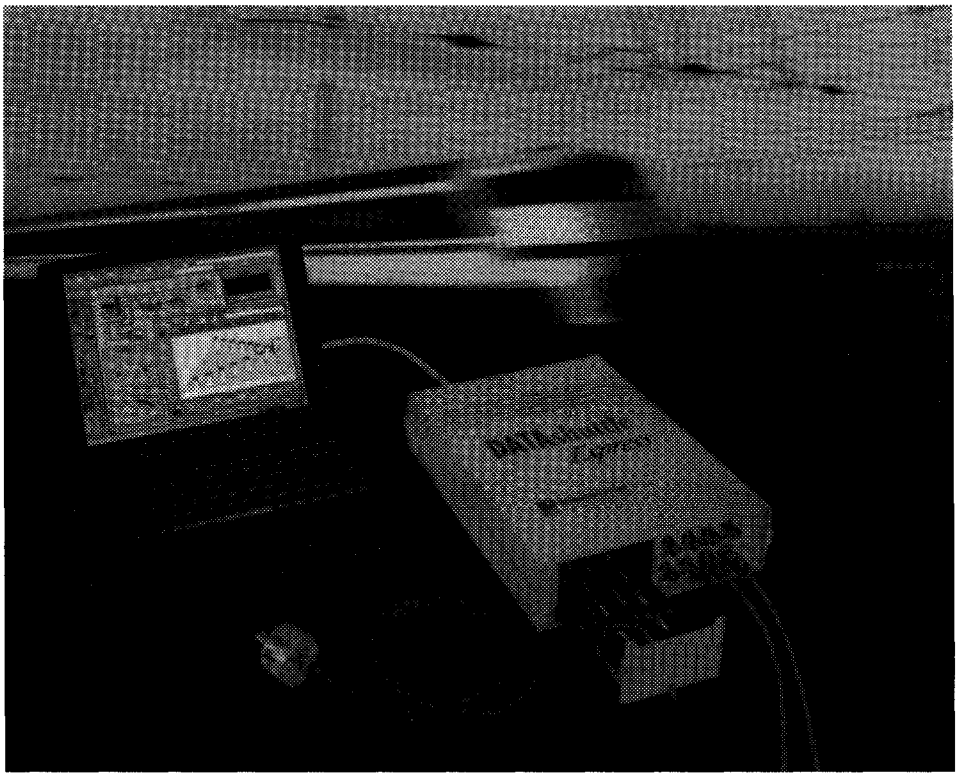

*The new DATAshuttle Express, a single-box data acquisition device, which allows high speed measurements to be taken from a variety of transducers and sensors using a standard notebook or desktop PC. The portable DATAshuttle Express incorporates transducer excitation and simply plugs into a PC's parallel port. Details from Adept Scientific, 6 Business Centre West, Avenue One, Letchworth, Herts SG6 2HB, UK. Tel.: 01462 480055.*

### Centrifugal Liquid Extraction (CLE)

The VectaSep CLE® Extraction device, recently launched by Whatman International, has quickly established itself as a time and cost saving means of preparing aqueous clinical samples prior to chromatography or other analytical techniques. A good example of its effectiveness is in the analysis of Ibuprofen in urine, where 95–100% extractions can be achieved when present at levels of 1–5 μg/ml. The ready-to-use VectaSep CLE® is made from precision moulded, high purity polypropylene; it is a disposable sample extraction device and has been created for use in 15 ml rotor cups within standard laboratory centrifuges. It comprises four parts: a tube with separate cap, a sample disperser with integral microporous dispersion membrane which forms the basis of the controlled centrifugal extraction process and a separating cup.

Extraction of the Ibuprofen from urine is by a simple two-step process. The first step involves the controlled extrusion of a 500 μl urine sample, diluted to 1·5 ml with 0·02 M phosphate buffered saline containing the internal standard Flurbiprofen, from the sample disperser through the dispersion membrane into 8·5 ml of extraction solvent (ethyl acetate). The extrusion requires just a short 10 minute centrifuge spin, undertaken at 35 °C and 1500 g.

In the second stage, the sample disperser is replaced by a separating cup. A second, 10 s centrifuge spin locates this cup at the solvent/sample junction, prior to the selective isolation of the Ibuprofen and the internal standard by evaporation to dryness under a light stream of oxygen free nitrogen, at 50 °C.

Finally, the extract is reconstituted in 70% acetonitrile and 30% 0·05 M phosphoric acid and injected onto a

0142-0453/97 $12·00 © 1997 Taylor & Francis Ltd

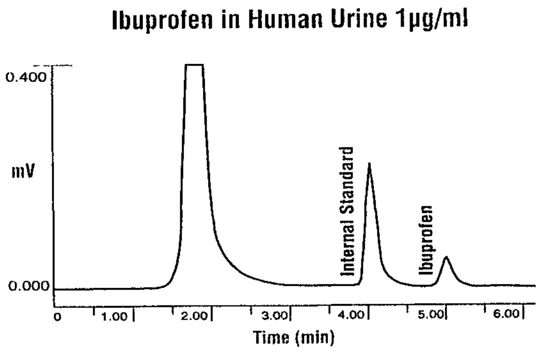

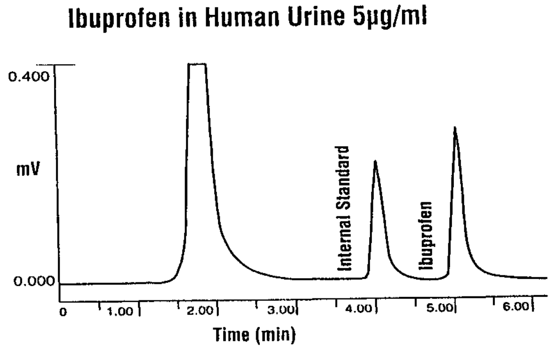

*Chromatograms of Ibuprofen in human urine at two concentrations compared with an internal standard of Flurbiprofen. Samples prepared using VectaSep CLE.*

PartiSphere C18 chromatography column. The analytes can then be detected using UV.

*Details from Whatman International Ltd, Whatman House, St Leonard's Road, 20/20 Maidstone, Kent ME16 0LS, UK. Tel.: 01622 676670; fax: 01622 677011.*

### CFAs

The processing power of Bran + Luebbe's CFA systems (continuous flow analysers) has been extended with the introduction of a new software program: AACE for Windows 95. Designed by chemists specifically for CFA systems, AACE manages the system via the control interface linking the analyser to the PC. Other Windows applications can run in the foreground or background during the analysis process. Windows 95's pre-emptive multi-tasking ensures that the analyser programs always receive the correct priority. In addition, it allows networking and two-way data transfer using the standard Windows 95 and Windows NT even during the analysis cycle. Future laboratory requirements have been taken into consideration as Windows 95 enables two completely independent analysers with up to 16 separate channels to be run, whilst larger installations can be controlled with Windows NT.

With AACE for Windows 95, users of Bran + Luebbe TrAAcs and AutoAnalyzers II instruments can run several different systems with a variety of samplers simultaneously on the same PC, plus down-load and up-load data from and to the laboratory management system.

*For further information contact: Bran + Luebbe Ltd, Scaldwell Road, Brixworth, Northants NN6 9UD, UK. Tel.: 01604 880751.*

### Zinc plating thickness on steel measurement

Oxford Instrument's Industrial Analysis Group has recently been awarded a US patent for the technology of its Lab-X 3000, the benchtop EDXRF (Energy Dispersive X-ray Fluorescence) Spectrometer. This analyser has a separate autosampler and uses Oxford's Focus-5 technology to increase sensitivity. It is capable of detecting elements from aluminium to uranium, from ppm to high %; but, it does not use radio-isotopes, hazardous chemicals or complex dilutions.

Several dedicated applications packages are also available which cover common uses including the determination of zinc plating thickness on steel. Samples are analysed 'as is', with little or no preparation; in this case of 40 mm diameter samples of the steel roll testing is usually complete in 60 s.

The autosampler allows for unattended operation for both routine analysis of samples and restandardization of a calibration line. Solid, liquid, and powder analysis can be undertaken using Oxford sample holders, combined with the corresponding sample tray. A 12-position, removable sample tray, robotic arm for transfer of samples and associated electronics control module can easily be operated by both lab and non-lab staff in quality control or research & development. The unit is situated on top of the Lab-X3000 and does not require additional benchspace. The resident Analytical Software Package, ASP3000, ensures full software control and simplicity of use.

*Details from Oxford Instruments' Industrial Analysis Group at 130A Baker Avenue Ext., Concord, MA 01742, tel.: 800 447 4717 or 19/20 Nuffield Way, Abingdon, Oxon OX14 1TX, UK, tel.: 01235 532 123.*

### Environmental analysis using high resolution EDXRF

Oxford Instruments' Industrial Analysis Group's Energy Dispersive X-ray Fluorescence (EDXRF) spectrometer, the ED2000, offers rapid non-destructive analysis of solids, liquids, powders, and slurries for up to 50 elements, with full composition of the sample available in minutes. The analyser offers a suitable alternative to AA or ICP for environmental analysis applications, including the measurement of toxic heavy metals and halogens in liquid hazardous waste; trace elements in soil and recycled materials, such as electronic/plastic waste; and petrochemical catalysts.

Developed in conjunction with users in the waste industry, the QuickSolve package from Oxford simplifies the analysis of liquid hazardous waste and meets the new ASTM method 6052. It allows rapid measurment of samples such as aqueous or organic solvents, sewage sludge, oil and water mixes etc., using one calibration line. Soil analysis is carried out with equal ease and detection limits of sub-ppm for trace heavy metals such as cadmium are achieved. Detection limits for the majority of transition elements are below 10 ppm, for example, with chlorine from waste oils down to a few ppm. System operation is controlled by XpertEase, Oxford's XRF Window™ software, which incorporates the pre-loaded methods. Qualitative, semi-quantitative, and full quantitative analysis techniques are all included, plus an 'unknowns' facility, which gives rapid elemental composition of samples with the results presented in a powerful graphical display. The software also allows a database of results to be compiled, which can be used for future comparative work and report generation. Ease of use and total flexibility are ensured. The analyser offers exceptional results and total flexibility to cope with changing requirements in quality control, development and investigative problems.

*Details from Oxford Instruments (see above).*

### X-ray fluorescence analysis of petroleum products

The MDX1000 uses a new technique in X-ray fluorescence-multi-dispersive XRF. The technique combines the benefits of the classical techniques of EDXRF and WDXRF to allow high performance, simultaneous measurement to be made on both low and high atomic number elements.

Oxford Instruments' MDX1000 is a fully integrated spectrometer which was designed with the industrial user in mind. It can be located either in a laboratory or close to the process to be controlled. The spectrometer's detection system allows simultaneous measurements to be made over a wide concentration range, from high percentage to ppm levels in a variety of sample types—including liquids, solids, granules, powders and pastes.

Dedicated applications packages, including ones designed for routine analysis of sulphur in oil to ASTM D2622; lead in petrol to ASTM D5059; nickel and vanadium in fuel oils; and magnesium, potassium, sulphur, chlorine, calcium, barium and zinc in lubricating oils are available. Additional applications packages designed for analysis of minerals, limestone, cement, glass and silica sand, and polymers are also available.

*More information from Oxford Instruments (see above).*

### Free spectra and chromatographic data viewer

The Galactic Data Viewer is a tool for viewing and displaying spectra and chromatographic data in the popular Galactic SPC format. It supports zooming, autoscaling, viewing single subfiles in a multifile, and copying images to the clipboard. The program, which runs under Windows 95 or Windows NT4·0, is provided free of charge and can be down loaded from the Galactic web site—http://www.galactic.com.

A beta test copy of an enhanced version of the Galactic Data Viewer is also available on the web site. In addition to the features of the standard viewer, the enhanced version supports OLE linking and embedding. This OLE compliant server application allows users to copy and embed spectral traces or chromatograms into any OLE compliant application and view/zoom the data in place. The enhanced version is available for free trial use and the final version will be available for a nominal charge.

### Veterinary drug residue analysis

*LC-MS Analyses of Veterinary Drug Residues using a Bench-Top Mass Detector* describes methods of drug residue LC-MS analysis using atmospheric pressure chemical ionization (APcl) and electrospray (ES).

Antibiotics and anthelmintics are amongst the wide range of veterinary products used to treat farm animals during outbreaks of disease or as a preventative measure when modern intensive practices are used. To reduce any possibility of harmful levels of such compounds reaching the human food chain, Maximum Residue Limits (MRLs) have been set up by the EU and many other countries, and are policed by regulatory bodies. Other growth-promoting drugs, including anabolic steroids, thyreostats and $\beta$-agonists, may be administered by farmers as a means of attracting carcass premiums when animals are presented for slaughter. Use of these substances to promote growth is banned within the EU, a ban which also requires policing. Thus, laboratory testing of food products must ensure that all regulations are met. For many of these compounds there is a wide range of available screening tests. However, for regulatory purposes, more sophisticated procedures are needed to confirm positive results from these tests. The recommendation is that confirmatory analyses should use mass spectrometry (MS) wherever possible. Many of the compounds are suited to gas chromatograph (GC)-MS, although some are difficult to analyse by this method, in which case liquid chromatography (LC)-MS may offer a more suitable alternative.

With many years of experience in determining drug residues using LC-MS, Micromass initially began working with Thermospray (TSP) techniques and more recently, moved on to APcl and ES. Methods developed using TSP have generally been found to perform better than APcl.

*Micromass can be contacted at Floats Road, Wythenshawe, Manchester, M23 9LZ, UK. Tel.: 0161 945 4170; fax: 0161 998 8195; Internet: http://www.micromass.co.uk.*

### Energy-saving processor

At just 10 × 15 × 7 cm, the compact VacuProcessor is compact and simple to instal and was designed for use in process industries employing vacuum technology. Many multi-suction cup systems can be over dimensioned as designers try to compensate for leakages caused by poor or non-attachment of suction cups. In most cases, this is dealt with by increasing the underlying vacuum. As a result, systems often become complex and expensive as pumps are added to meet vacuum requirements. Available in two versions, electronically (MCE 4-001) and pneumatically (MCP 4-R) regulated, the VacuProcessor was designed to solve these problems and a single unit can control up to four channels. Each channel has its own pre-set vacuum guard connected to a control valve. In the event of a leakage, the unit automatically switches off the corresponding valve.

In the electronically regulated version, suction cup operation can be defined to meet complex handling requirements. In simple terms, should only three cups be needed, then the fourth is switched off. In systems where more than four suction cups are required, a number of VacuProcessors can be connected to create a multi-cup system.

Further energy savings are possible by installing the VacuProcessor together with an ES-08/1 or ES-25/1 energy saver system. This is a simple, dial controlled unit which maintains a stable vacuum without the need to constantly run the pump which is only switched on when the vacuum drops below a pre-set level.

*Details from International News Service—INS AB, S-131 84 Stockholm, Sweden. Tel.: 46 8 601 00 00; fax 46 8 718 45 90. E-mail: webmaster@ins.se.*

### Preservation of the science base

Sir Aaron Klug O.M., president of the Royal Society, considers the primary role of the Society to be 'preserving the health of the science base at a time when the prevailing philosophy, not only in the UK, is that public sector activities should be subjected to market forces, which would see to it that sound policies emerge'. In his presidential address at the end of 1996, he highlighted the Society's capability to provide independent, authoritative advice, notably to government, on science and engineering-related matters, and to inform public debate.

The Royal Society has, for example, been important in 'getting to grips with the problem of BSE ... When the BSE/CJD crisis broke in March 1996, the Society was able to harness a formidable group of Fellows and outside experts on all aspects of the problem. This group met again in June 1996 when there were indications that BSE might have been passed on to sheep. The Society produced a series of clear and significant statements, steering clear of political sensitivities, which we circulated widely and which helped to explain the situation. The BSE Group has been kept in being since there are now further developments, particularly recent biochemical evidence that the new variant form of CJD is related to BSE'.

Another major task for the Society is considering the Conservative Government's Prior Options Review process for the possible privatization of public sector research establishments in the UK: '43 establishments are under review and the exercise is being conducted in what appears to be unseemly haste. Logically there are very good reasons for examining the mission of a PSRE, and in certain areas, and for certain institutes, privatization may be the right solution. But it is very evident that this repeated examination is doing damage in terms of productivity and morale. Indeed, the sensible way to proceed would be for all PSRE's to be subject to periodic external reviews (say every five years) of the type carried out by Research Councils who use visiting committees, which include international experts where necessary'. He stressed the importance of basic and strategic research and research which is essential to underpin the statutory, policy and regulatory functions of government, such as that conducted in the Toxicology Unit of the Medical Research Council or in the Public Health Laboratory Service. He said that research is also needed to underpin other functions of government, such as defence and health—a long-term need to build up expertise in readiness for unexpected developments.

'Basic research can ultimately yield extraordinary returns to society, but it is difficult to estimate its benefits quantitatively since its results may be used in many directions. Moreover there is often a significant delay between the dissemination of fundamental knowledge and its eventual effect on industrial processes. Basic investment must be made many years ahead and the investment must be not only in institutions but above all in people, in human resources. Research may be initiated in programmes and by committees, but it takes place in the brains and hands of individuals'.

The discovery of a new form of carbon, closed spherical cages of carbon atoms called fullerenes, made by a Royal Society Research Professor, Sir Harry Kroto, F.R.S., and two American colleagues, arose from 'as unlikely a quarter as one could imagine, namely Kroto's research to investigate compounds of carbon in interstellar space'. This has led to further discoveries with new possibilities for superconducting materials. The discovery was also recognized this year by the award of the Nobel Prize for Chemistry.

*A copy of Sir Aaron's full speech is available from the Press & Public Relations Unit, The Royal Society, London SW17 5AG. Tel.: 0171 451 2516/7.*

